# Human Subcutaneous Dirofilariasis Behind a Blepharoconjunctivitis: A Case Report and Review of the Literature

**DOI:** 10.7759/cureus.60208

**Published:** 2024-05-13

**Authors:** Sergiu Morosanu, Roman Don, Valentin Morosanu

**Affiliations:** 1 Cardiology, Targu Mures Institute for Cardiovascular Diseases and Heart Transplantation, Targu Mures, ROU; 2 Ophthalmology, Emergency Hospital Professor Doctor Nicolae Oblu, Iasi, ROU; 3 Neurology, Emergency County Hospital Targu Mures, Targu Mures, ROU

**Keywords:** eyelid involvement, subcutaneous dirofilariasis, heartworm, uncommon etiology, blepharoconjunctivitis

## Abstract

The diagnosis of skin lesions involving the eyes can be challenging, especially when uncommon etiologies are considered. We present a case of a 52-year-old female initially diagnosed with blepharoconjunctivitis but later found to have a subcutaneous heartworm infection. The patient experienced recurrent episodes of unilateral palpebral edema, pain, pruritus, and a sensation of a foreign body in her eye. Upon examination, a vermiform structure with peristaltic movements was observed, raising suspicion of subcutaneous dirofilariasis and prompting further investigations. Serological tests confirmed the presence of anti-*Dirofilaria* spp. antibodies. Surgical removal of the worm led to the resolution of symptoms. This case highlights the importance of considering uncommon etiologies, such as subcutaneous heartworm infection, in patients presenting with atypical migratory skin lesions or ocular manifestations when there is no definite diagnosis and the condition does not respond to usual medical treatment.

## Introduction

The etiological diagnosis of primary and secondary skin lesions can be particularly challenging in certain cases, especially when the eyes are involved. Regardless of the causative agent, the local response to aggression typically remains similar, and once we rule out the most common etiologies, we must consider less frequent causes. Here, we present a rare case of a 52-year-old female who was initially diagnosed with blepharoconjunctivitis but was later found to have a subcutaneous heartworm infection.

Subcutaneous dirofilariasis, caused by infection with species of the *Dirofilaria* genus, presents a diagnostic challenge due to its nonspecific clinical manifestations and low incidence in humans [1]. The infection most frequently affects carnivorous animals, especially canids and felines, with human infection being primarily incidental. The most demanding aspect is prompting clinical suspicion of heartworm disease, following which diagnosis and treatment procedures tend to become relatively straightforward [2-4].

## Case presentation

**Figure 1:**
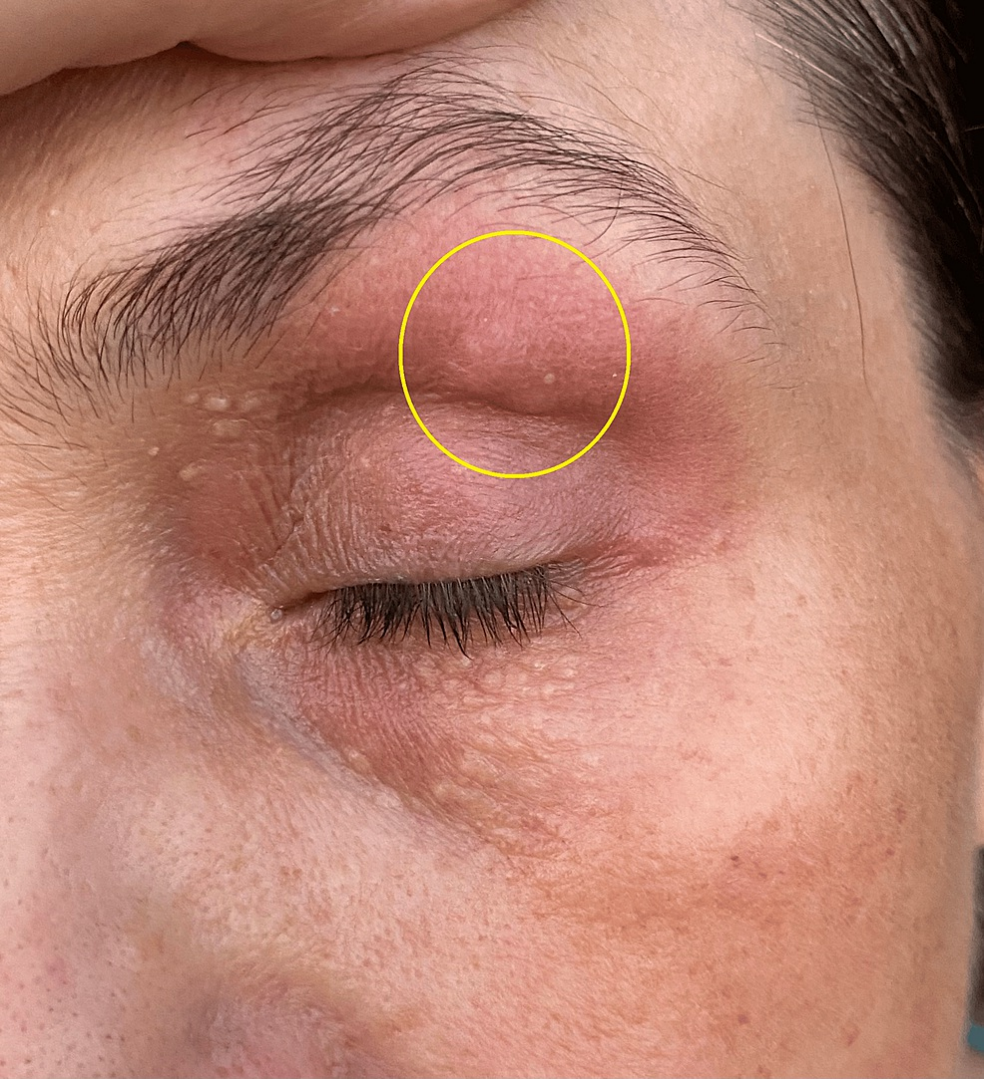
Erythematous papular lesion in the left palpebral region.

A 52-year-old female complained of recurrent episodes of unilateral palpebral edema accompanied by intense paroxysmal pain, pruritus, local flushing, and a foreign body sensation affecting both of her eyes consecutively. These symptoms lasted for minutes to hours, had sudden onset and spontaneous disappearance, and showed an inconsistent relationship with the use of cosmetics. The symptoms initially occurred in the right eye (RE) and later affected the left eye (LE), with onset a month earlier. She had no significant medical history and had not traveled in the past five years. She lived in a developing country in Eastern Europe and owned a cat and a dog that had not been properly dewormed. The complete clinical examination at the very first medical contact revealed a discrete erythematous and pruritic papular lesion in the left palpebral region, inflammation of the meibomian glands, tarsal and bulbar conjunctival hyperemia, and a mild mucopurulent discharge suggesting blepharoconjunctivitis (Figure 1).

The complete blood count and serum biochemistry had shown only mild eosinophilia (800/µL), which spontaneously resolved afterward. Workups for inflammatory markers, thyroid dysfunction, and allergy profiles, including Prick tests for the most common allergens, were all negative.

The patient was advised to avoid eye makeup, and topical treatment with corticosteroids, tobramycin, and moxifloxacin, along with systemic antihistaminic treatment with loratadine, was initiated. However, there was no significant clinical improvement. More than that, she had noticed a cord-like, vermiform structure with its own discrete mobility corresponding to the palpebral swelling for two days prior to re-evaluation.

**Figure 2:**
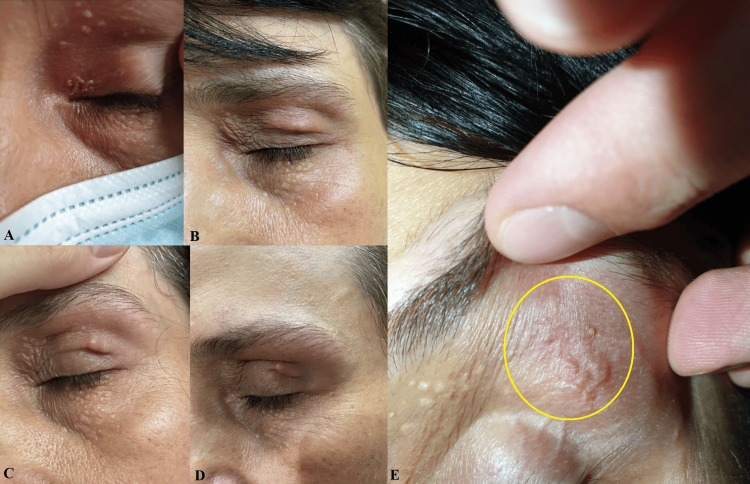
Migration of the lesion during a 24-hour follow-up. (A)-(E) Lesion migration from the inner corner of the superior eyelid to the lateral eyebrow.

The clinical examination at re-evaluation revealed a vermiform structure measuring approximately 2/20 mm, exhibiting slow, peristaltic-like movements and accompanied by local swelling and flushing. In the pictures below, we can observe the migration of the lesion from the internal to the external palpebral angle during a 24-hour follow-up (Figure 2).

Given the clinical presentation at that time, especially the lesion's migratory behavior over a short span and its autonomous movement, common diagnoses such as tumors, granulomas, cysts, or abscesses became less likely. This directed our focus toward potential subcutaneous parasitic infections, including those caused by *Dirofilaria*, *Loa loa*, *Onchocerca*, *Brugia*, *Spirometra*, *Sparganum*, or others. Consequently, it was decided to perform an enzyme-linked immunosorbent assay (ELISA) test to detect antibodies corresponding to *Dirofilaria* spp., *Brugia* spp., *Loa loa*, *Mansonella *spp., *Onchocerca* *volvulus*, and *Wuchereria* *bancrofti*. The results revealed positive IgG-type antibodies against *Dirofilaria immitis*, as expected, considering the geographical area where the patient lived and the absence of trips abroad.

Due to the intermittent nature of signs and symptoms, the patient was advised to seek immediate consultation with the ophthalmology department once the structure became visible again, with the intention of attempting surgical removal. The clinical examination findings were as follows: visual acuity (VA) in the RE was 5/9, and in the LE was 5/6, consistent with her medical history. Intraocular pressure (IOP) was 14 mmHg in the RE and 15 mmHg in the LE. The anterior segment of both eyes showed inflammation of the meibomian glands, tarsal and bulbar conjunctival hyperemia, and mild mucopurulent discharge at the inferior conjunctival fornix, more pronounced in the left eye and slight in the right eye. No other pathological findings were observed, including upon examination of the posterior eye segment and extraocular muscles. Subsequently, a small palpebral incision was made, and the worm was extracted using forceps. Histological examination findings were consistent with an immature female worm of *Dirofilaria*, but no further molecular diagnostic tests were conducted to define the species. The postoperative course was favorable, with no recurrence of symptoms and no need for additional medical treatment.

## Discussion

Dirofilariasis is a disease caused by infection with species of the Dirofilaria genus, which belongs to the class of nematodes and is transmitted by vectors. It mostly affects canids and, less frequently, felines, raccoons, and other mammals, causing cardiopulmonary or subcutaneous dirofilariasis. Sporadic human infections have also been reported. Moreover, seroprevalence studies indicate that the rate of infection in humans is almost similar to that in canids and felines in endemic areas, suggesting that a significant portion of human infections are asymptomatic or self-limiting [5-9].

The species most frequently involved in human infections are *D. repens*, *D. immitis*, and *D. tenuis *[4]. Both *D. immitis* and *D. repens* cause symptomatic infections in canids and felines, with the former responsible for cardiopulmonary infections and the latter for subcutaneous ones, although there are exceptions to this rule. In the case of *D. tenuis*, the primary host is mainly the raccoon. Human subcutaneous or pulmonary dirofilariasis can be caused by any of the aforementioned parasites [3,4,10].

The life cycle of the parasite throughout the course of subcutaneous dirofilariasis is well understood. The vector, represented by a female mosquito of the *Culicidae* family, deposits the infectious larvae of *D. repens* onto the host's skin during a blood meal, followed by the larvae penetrating the skin on their own. In the subcutaneous layer of the skin, the larvae undergo a maturation process and begin producing microfilariae that are released into the bloodstream and ingested by mosquitoes during a subsequent blood meal. Inside the mosquito's body, the microfilariae develop into infectious larvae and migrate to the mosquito's mouthparts, thus setting the stage for infecting a new host. The life cycle is similar in the case of pulmonary dirofilariasis, except that adult parasites producing microfilariae are located inside the pulmonary arteries [3,4].

Typically, humans do not serve as definitive hosts for *Dirofilaria*, so the majority of reported cases involve infections with immature worms, which are incapable of producing microfilariae, as was the case with this patient as well. However, complete growth and multiplication of *Dirofilaria* in humans cannot be ruled out, as there are cases of subcutaneous dirofilariasis with mature females and significant microfilaremia described in the literature [11-13].

From a clinical perspective, human dirofilariasis presents as two distinct entities. The first is most commonly associated with *D. immitis*, whose larvae can withstand local immune responses in the subcutaneous layer of the skin. They migrate into the systemic venous circulation and then to the pulmonary arteries, where they can cause small zones of pulmonary infarction and where they are neutralized by the immune system and incorporated into a granuloma, primarily composed of eosinophils, lymphocytes, and plasma cells. Clinically, this phenomenon manifests as a nodular, coin-like lesion detected on chest radiographs or computer tomography (CT) scans, posing a challenge in differential diagnosis with neoplastic lesions [3,14].

The second entity, caused more frequently by *D. repens*, is characterized by subcutaneous and/or ocular manifestations due to parasite migration and local immune responses. It can present as a localized or migrating subcutaneous nodule, accompanied by local inflammatory signs. In cases of ocular dirofilariasis, involvement of orbital, periorbital, subconjunctival, or intraocular tissues may lead to visual disturbances such as loss of sight, palpebral ptosis, edema, blepharitis, conjunctivitis, and other manifestations secondary to possible local complications. The differential diagnosis of these lesions can be quite challenging, and due to the low incidence of dirofilariasis in humans, clinical suspicion of heartworm infection typically arises late in our consideration when dealing with these patients [3,14,15].

The definitive diagnosis of dirofilariasis can only be established by evaluating the morphological features of excised tissue and/or extracted parasites. Serological techniques such as ELISA or Western blot are available and can be helpful in the differential diagnosis. However, their cross-reactivity with different species of *Dirofilaria* and other helminths, as well as the high seroprevalence of anti-*Dirofilaria* spp. immunoglobulins in endemic regions, limit their usefulness. Additional molecular diagnostic tests are needed to define the particular species of *Dirofilaria* [16-18].

The treatment for human dirofilariasis typically involves surgical removal of the lesion in the vast majority of cases, with no additional medical treatment necessary. However, if microfilaremia is present, systemic antifilarial agents such as ivermectin or doxycycline should be considered [19,20].

## Conclusions

In conclusion, dirofilariasis remains a rare but notable condition that poses diagnostic challenges due to its varied clinical presentations and low incidence in humans. This case underscores the importance of considering uncommon etiologies, such as subcutaneous heartworm infection, particularly in patients with atypical skin lesions or ocular manifestations. The most crucial stage in approaching these patients is raising clinical suspicion of dirofilariasis, which is favored by the migratory nature of the lesion or, moreover, by the peristaltic movements of heartworms at the subcutaneous level observed during the clinical examination. In the absence of these clinical phenomena, the diagnosis is quite challenging, as heartworm infection is often one of the last conditions considered. In this case, the paraclinical examinations that come to our aid to strengthen our diagnostic suspicion are serological tests, polymerase chain reaction (PCR), or imaging investigations that describe the morphological properties of the lesion and the neighboring tissues, including high-resolution ultrasonography, computed tomography, or magnetic resonance imaging. However, none of these provides us with a definitive diagnosis.

A particular scenario arises when the patient reports visualizing the movement of the parasite at the subcutaneous level despite the absence of objective evidence. Stigmatization and labeling of symptoms as delusional parasitosis, known to the general public, must be vehemently avoided because, as seen in our case, both the symptoms and clinical signs can vary from day to day and even hourly. 

Once we consider all of this, both diagnosis and treatment become relatively straightforward, with an excellent long-term prognosis. This is primarily due to the increased recognition of the disease resulting from a higher incidence of infections, as well as from the rising number of case reports published, especially in the last two decades. A future challenge would be the possibility of achieving a definitive diagnosis through non-invasive methods. This would facilitate the avoidance of surgical interventions in cases with difficult approaches, especially in asymptomatic patients with incidentally found lesions where the risk of surgical complications is higher than that of the infection itself.
